# Geographical differences in the use of oral corticosteroids in patients with severe asthma in Spain: heat map based on existing databases analyses

**DOI:** 10.1186/s12890-022-02295-2

**Published:** 2023-01-04

**Authors:** Carlos Almonacid, Eunice Fitas, Joaquín Sánchez-Covisa, Héctor Gutiérrez, Pablo Rebollo

**Affiliations:** 1 Pulmonology Unit, Complejo Hospitalario de Toledo, Av. del Río Guadiana, 45007 Toledo, Spain; 2 AstraZeneca Farmacéutica, Madrid, Spain; 3 IQVIA Spain, Madrid, Spain

**Keywords:** Severe asthma, Oral corticosteroids, Real-world data, Spain

## Abstract

**Background:**

Although there are currently alternative treatments to the long-term use of oral corticosteroids (OCS) in severe asthma, recent studies show excessive use depending on geography and differences in medical practice. The objective of the study was to describe the differences in OCS use for severe asthma across the Spanish geography.

**Methods:**

This is a real-world study using existing databases (year 2019): longitudinal patient database (EMR), based on electronic medical records, and database of pharmacological consumption (Sell-in) in basic healthcare areas. With EMR, the percentage of OCS prescriptions corresponding to patients with severe asthma (ICD-9 “asthma” and prescription of biological treatment and/or high dose of inhaled corticosteroids/long-acting inhaled β2 agonists) was calculated. This percentage was transferred to the OCS consumption of each basic healthcare area as reported in the Sell-in database and a national heat map was created. The estimation of OCS use in patients with severe asthma per 100,000 inhabitants for each region was calculated by grouping basic healthcare areas and the mean OCS use per patient for different regions in Spain was also estimated.

**Results:**

Patients with severe asthma in Spain were mostly female (69.6%), with a mean age (SD) of 57.6 years (18.01). Median time (Pc25–Pc75) since asthma diagnosis was 83.1 months (34.65–131.56). Of all patients with OCS prescriptions in 2019 identified in EMR, 4.4% corresponded to patients with severe asthma. Regions with the highest OCS use were Asturias, Andalucía, and Galicia, whereas those with the lowest use were Navarra, Baleares, Madrid and País Vasco. The mean OCS use per patient with severe asthma in 2019 throughout Spain was 1099.85 mg per patient, ranging from 782.99 mg in Navarra to 1432.64 in Asturias.

**Conclusions:**

There are geographical differences between Spanish regions with respect to the use of OCS in patients with severe asthma. The national mean consumption of OCS per patient with severe asthma and year is above the limits that indicate good asthma control.

## Background

Asthma is a common and potentially serious chronic inflammatory respiratory disease with a substantial burden on patients, their families, and the community. It causes respiratory symptoms, limitation of activity, and exacerbations that sometimes require urgent health care and might have fatal consequences [1]. The prevalence of asthma is higher in industrialized western countries (10%) compared to the underdeveloped countries (≤ 1%), although this may be due to an underestimation of cases in the less developed health systems [2]. Within western countries, its prevalence is higher in urban areas than in the rural population [2]. It has been estimated that approximately 5–10% of asthma patients have severe disease [3]. Severe asthma is defined as uncontrolled asthma despite adherence with optimized high-dose inhaled corticosteroid (ICS); long-acting beta2-agonist (LABA) therapy and treatment of the contributory factors, or disease that worsens when high dose treatment is decreased [3–5].

The Global Initiative for Asthma (GINA) recommends a stepwise approach to asthma therapy, aiming to optimize symptom control and reduce the risk of exacerbations [1]. Despite these recommendations, patients with asthma remain at high risk of severe exacerbations due to the lack of adherence to maintenance therapy, overuse of reliever therapy, poor inhaler technique, comorbidities, or difficult-to-treat asthma [6]. Hence, many patients with severe asthma require two or more oral corticosteroid (OCS) bursts per year for managing their exacerbations [7]. According to a recently published analysis of the International Severe Asthma Registry, patients with severe asthma have an average of four severe exacerbations per year, and therefore, require four bursts per year on average [8]. Considering the adverse events associated with the use of OCS, recent guidelines allow their use as the last therapeutic alternative [4]. Furthermore, due to the higher risk of adverse effects, an expert consensus recommends tapering of OCS for the treatment of asthma, while focusing on the general objective of avoiding the use of OCS [9]. However, the use of both maintenance OCS and the repetitive bursts for acute asthma exacerbations lead to cumulative adverse events [10, 11] thereby, increasing the healthcare costs [12].

A systematic literature review showed that the use of long-term and repeated short-term oral/systemic corticosteroids were associated with an increased risk of acute (infections and gastrointestinal events) and chronic (metabolic, bone and muscle-related, cardiovascular events, psychiatric complications and ocular complications) adverse events as compared to the non-use, resulting in increased healthcare resource use and costs [13]. A pharmacoeconomic evaluation using real-life data from a registry in Italy showed that the annual cost per patient with severe asthma when compared to a non-asthma control cohort and a moderate asthma cohort, resulted in approximately €892 and €606 higher costs, respectively, further depicting a corticosteroids shadow cost ranging from 30 to 45% [14]. The budget impact model estimated a total annual cost of €242.7 million for OCS-related adverse events in patients with severe asthma. This model applied the cost per patient to the target population identified for Italy (N = 123,988 patients with severe asthma receiving OCS) to determine the total annual cost. Taking into account the social perspective, the economic impact of severe asthma in Spain was estimated to be €8554/patient/year [15].

A few years back, more than half of the patients with uncontrolled severe asthma required OCS to control their symptoms [7]. However, with the currently available new agents [16], along with multidimensional assessment [17], and the increasing use of selected biologics [18–20], the frequency of asthma exacerbations and the use of maintenance OCS can be reduced considerably, in patients with severe asthma. Although the recommendations for management of asthma have been updated—instructing cautious use of OCS add-on therapy due to side effects [1]—their extensive use in patients with severe asthma still continues, as indicated in several recent studies [11, 14, 21].

Recently, a prospective observational study (LEVANTE) conducted in Spain evaluated the use of OCS and the disease burden in patients with severe asthma who received the treatment in bursts and/or maintenance. The first published data [22–24] from 6 months follow-up showed the mean cumulative prednisone equivalent to be 1306.7 mg, while 30% of patients in maintenance required a mean of 2 OCS bursts. According to another published study [25], 21.7% of patients with severe uncontrolled asthma in specialized asthma units had treatment with OCS for 3 months or longer. These studies demonstrate a high variability in OCS treatment regimens across different geographical regions of Spain that could be due to differences in the clinical criteria of the professionals involved. However, the extent to which the differences in OCS use among patients with severe asthma are causing the undesired health consequences remains unclear, along with the variability in associated hospital admissions and costs among geographical regions in Spain.

This study aimed to describe the differences in the use of OCS in severe asthma across the Spanish geography by creating a national heat map.

## Methods

This is a real-world observational study using existing databases: *EMR database* and *Sell-in database*, provided by IQVIA corresponding to the year 2019. The year 2019 was chosen instead of 2020 because of the disruptive effect of COVID-19 on the healthcare activity in Spain. The *EMR database* is a large longitudinal database of electronic medical records that includes anonymized data collected from 1450 general practitioners (GPs) and 2000 specialists, representing around 1 million unique patients on an annual basis (~ 3.2% of the overall treated population in Spain). This database application assigns unique patient and doctor identifier codes to each user at a given center. Data encryption is ensured by a 128-byte algorithm that creates a non-reversible hash. Subjects included in the IQVIA’s *EMR database* have similar distribution by age and gender as compared to the Spanish general population. Data is projected to the national level based on the demographic attributes (age and gender) and covers both primary care and the outpatient specialty care.

The *Sell-in database* provides the information regarding drug uses (excluding hospital drug) per basic healthcare areas, which are the geographic areas (census sections) associated with a Primary Care Center. These basic healthcare areas can be aggregated into hospital reference areas and Autonomous Communities (AC). Spain is administratively divided into 17 AC (regions) and two autonomous cities included in the Andalucía AC in the present study, each with its particular health system. Drug uses were provided for the study without differentiating by indication.

The *EMR database* provided information on the use of OCS in severe asthma at the national level, whereas the *Sell-In database* provided the total OCS use (irrespective of the indication) in each basic healthcare area.

**Fig. 1:**
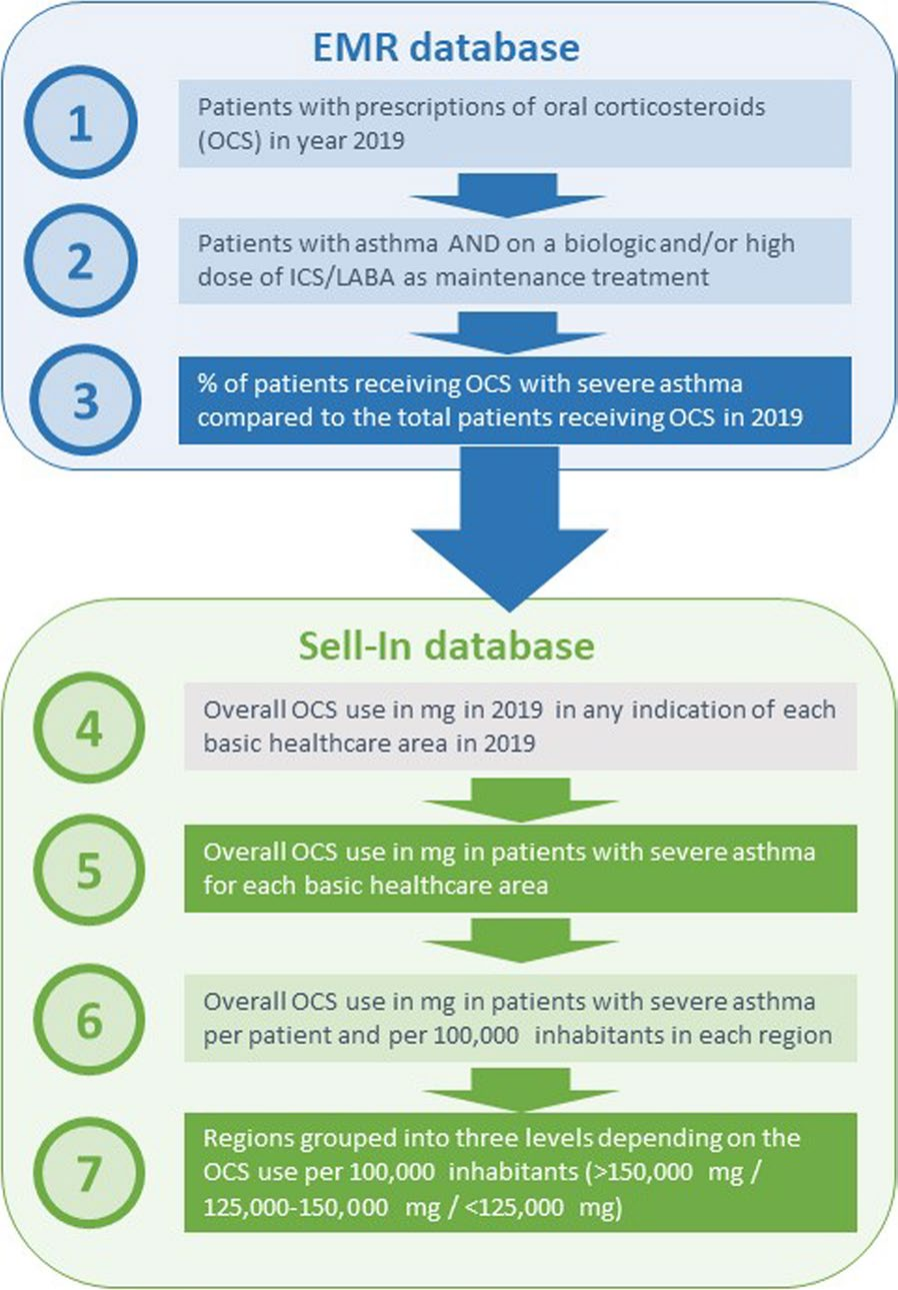
Use of the two databases (EMR database and Sell-In database) in the analysis

Initially, patients with at least one OCS prescription in 2019 were identified (ATC codes: H02AB13 deflazacort/H02AB07 prednisone) and quantified in the *EMR database* (year 2019). Among them, patients with ICD-9-MC diagnosis code for asthma (493.xx) were identified. Next patients with severe asthma were identified as those on a biologic and/or high dose of ICS/LABA as maintenance treatment according to ATC codes and dose definitions (Table 1). This allowed the estimation of the percentage of patients in 2019 receiving OCS with severe asthma compared to the total patients receiving OCS at the national level. Thereafter, the total OCS use in 2019 for any indication was obtained from the Sell-In database for each basic healthcare area across Spain. Finally, the OCS use in patients with severe asthma for each basic healthcare area of the *Sell-In database* was determined by applying the national percentage of patients with severe asthma receiving OCS, estimated in the first step, to the total OCS use of each basic healthcare area (Fig. 1). To avoid overestimation of OCS use, for 30-mg 30-tablets packs, it was assumed that only 30 mg twice a day for 5 days were used; the rationale behind this is that this was the only pack available in the dose strength recommended (20–40 mg every 12 h) by the guidelines to treat exacerbations that were in place in 2019 [26], but the usual physician’s instructions are to take it for 5–7 days.

**Table 1:** ATC codes and dose definitions used for identification of patients with severe asthma in the EMR database

Drug	ATC code	Pack dose
Omalizumab	R03DX05	Any
Mepolizumab	R03DX09	Any
Benralizumab	R03DX10	Any
Fluticasone/Vilanterol	R03AK	184/22
Beclometasone/Formoterol	R03AK	200/6
Fluticasone/Formoterol	R03AK	250/10
Budesonide/Formoterol	R03AK	320/9
Fluticasone/Salmeterol	R03AK06	500/50
Beclometasone/Salbutamol	R03AK13	50/100

Reslizusmab is not available in the EMR database

Hence, the outputs from both databases were combined to extrapolate the use of OCS in severe asthma in each different basic healthcare area. Using the decile distribution for total OCS (mg) use in patients with severe asthma per 100,000 inhabitants per year, color was assigned to each healthcare area to draw the national heat map with cold and hot areas regarding the use of OCS in severe asthma. 

The estimation of the use of OCS in severe asthma per 100,000 inhabitants for each region was estimated by grouping the basic healthcare areas. The mean OCS use per patient for each region and overall Spain was estimated following the steps below:

1. As it was explained above, the percentage of severe asthma patients with OCS prescriptions among all patients with OCS prescriptions using the *EMR database*.
2. By applying the percentage above to the total OCS consumption provided by the *Sell-in database* (at the national and regional level), the total OCS consumption among severe asthma patients for Spain and for each region was extrapolated.
3. The number of patients with severe asthma was estimated to be 0.5% of the general population: 10% of subjects from the general Spanish population have asthma [27] and 5% of these have severe asthma [5].
4. The number of patients with severe asthma who receive at least one OCS prescription was calculated by applying to the previous number (Step 3) the percentage of severe asthma patients who received at least one OCS prescription according to the *EMR database*.
5. Finally, the total OCS consumption among severe asthma patients was (numerator coming from Step 2) was divided by the number of patients with severe asthma who receive at least on OCS prescription (denominator from Step 4).

Additionally, the *EMR database* was used to describe the clinical and demographic characteristics of patients with severe asthma using OCS (age, gender, weight, height, body mass index, smoking status, time since asthma diagnosis and comorbidities). It was also used to estimate the national rate of OCS use among patients with severe asthma (mean and standard deviation).

## Results

According to the *EMR database*, patients with severe asthma in Spain were mostly female (69.6%), with a mean age (SD) of 57.6 years (18.01). The mean (SD) weight was 78.1 kg (19.53), height 160 cm (9.56), and the mean (SD) BMI was 30.49 (7.11). With respect to smoking status, 84.3% were non-smoker, and 10.2% were smokers (5.5% missing data). Median time (Pc25–Pc75) since asthma diagnosis was 83.1 months (34.65–131.56). The relative frequencies of selected comorbidities of interest in patients with severe asthma identified in the *EMR database*: rhinitis (36.8%), nasal polyposis (5.39%), gastro-oesophageal reflux (15.61%), chronic sinusitis atopy (28.81%), respiratory infections (30.77%), arrhythmia (2.86%), angina (3.05%), osteoporosis (11.80%), and arthropathy (9.14%).

**Fig. 2:**
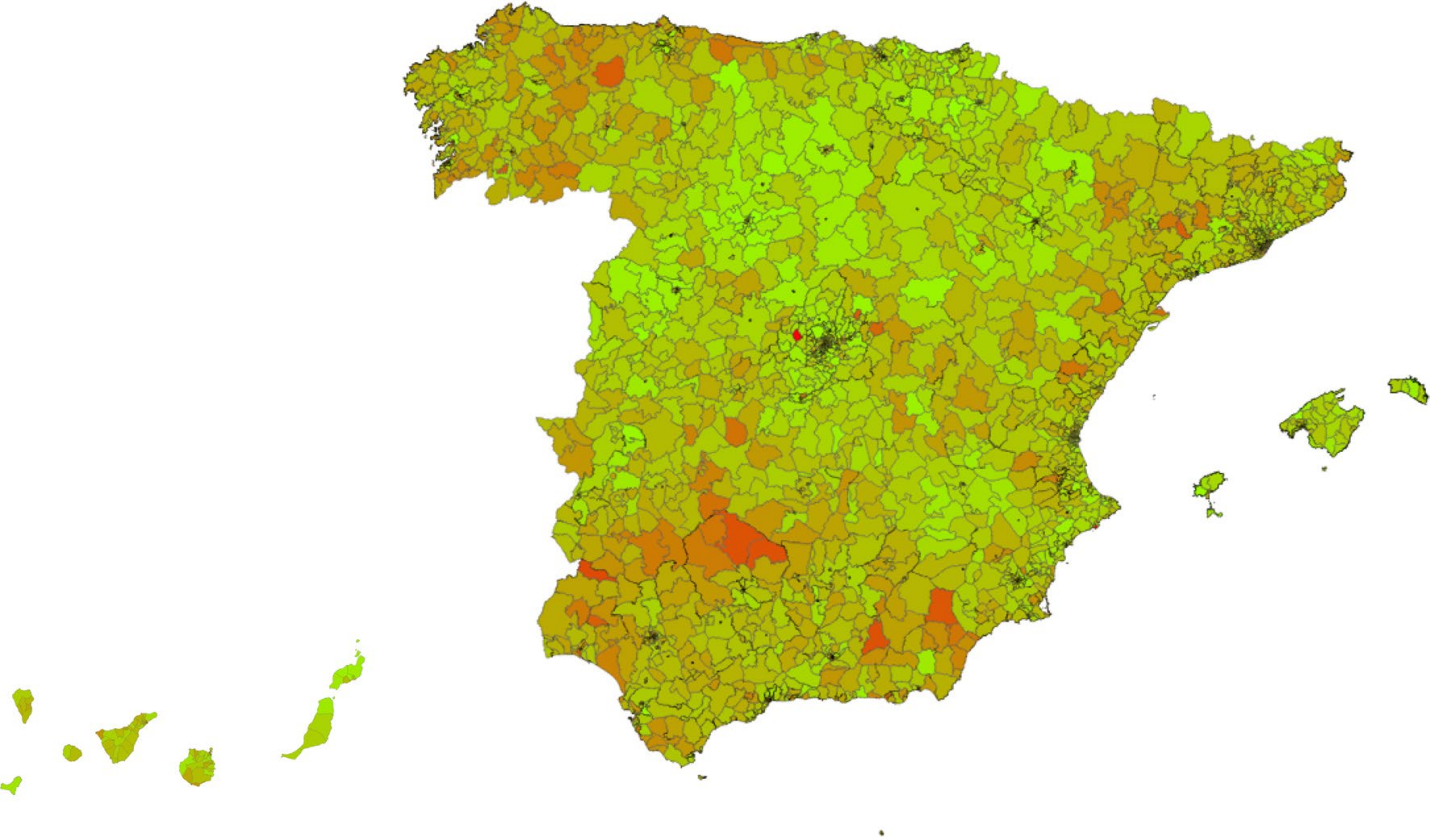
National heat map showing OCS use in patients with severe asthma (use per 100.000 inhabitants and per year). Red areas indicate highest OCS use, and the green areas indicate lowest OCS use (ranges defined according to the decile distribution)

**Fig. 3:**
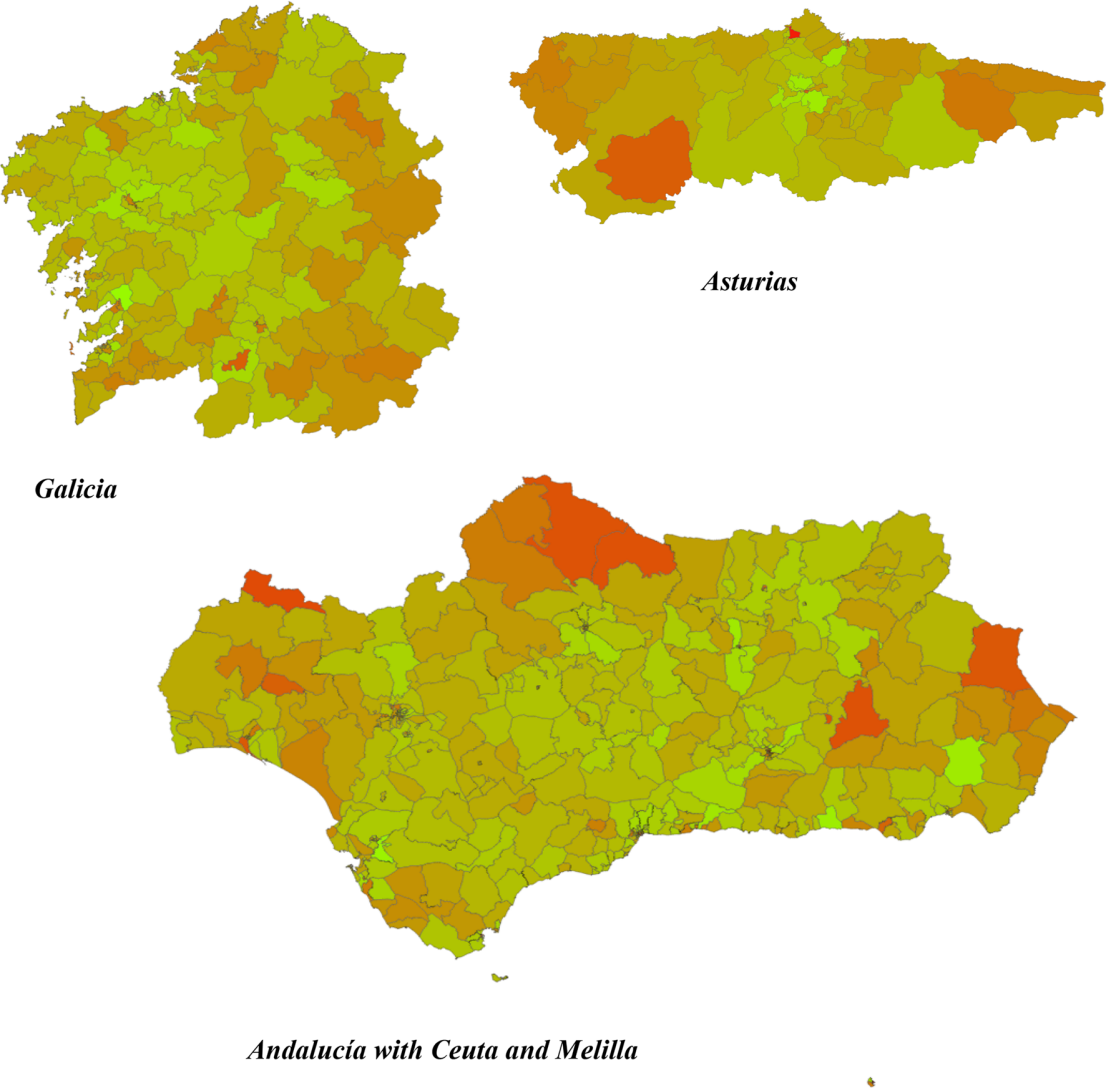
Regional heat map showing OCS use in patients with severe asthma (use per 100.000 inhabitants and per year) for regions with highest OCS use. Red areas indicate highest OCS use, and the green areas indicate lowest OCS use (ranges defined according to the decile distribution)

**Fig. 4:**
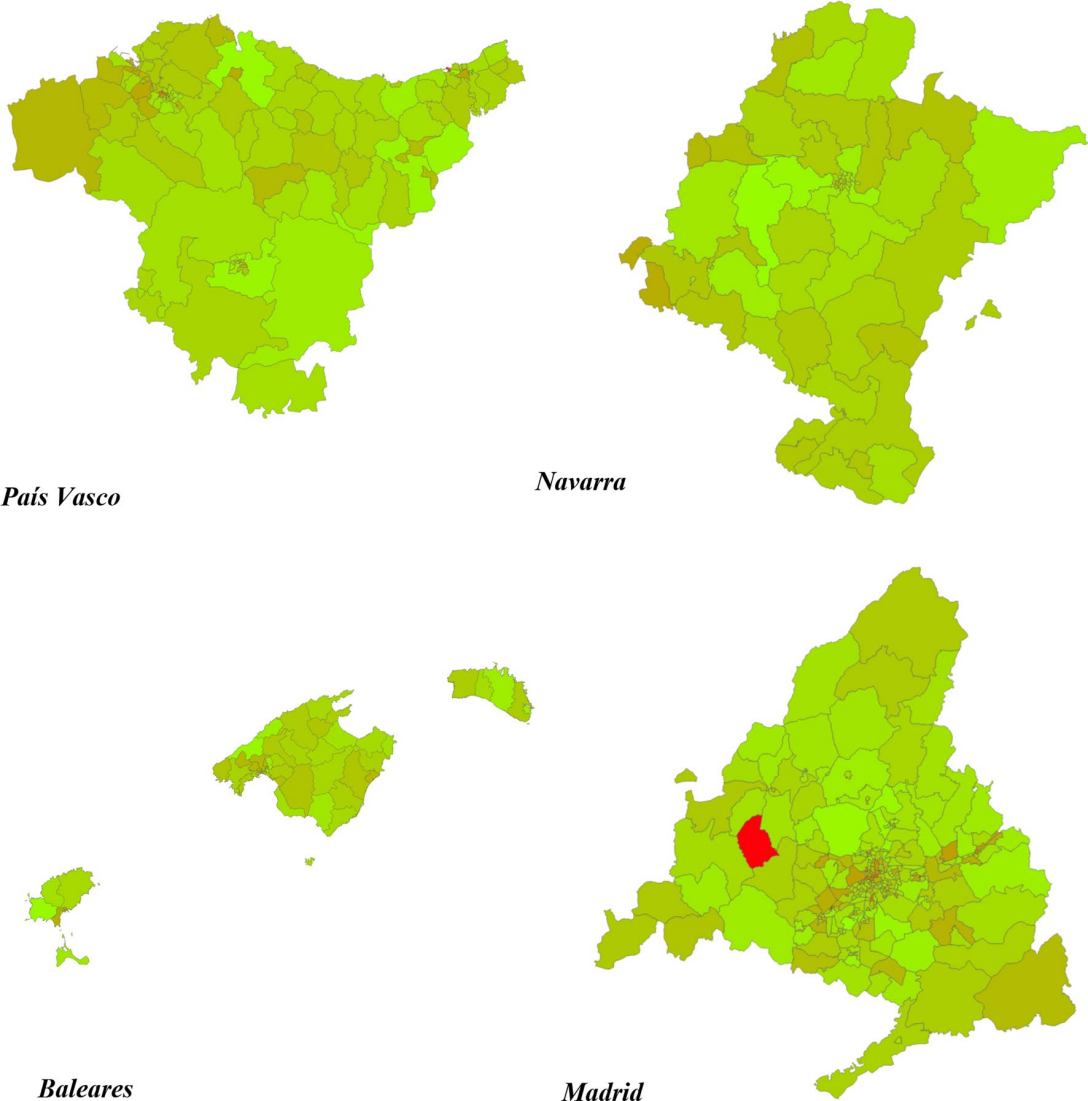
Regional heat map showing OCS use in patients with severe asthma (use per 100.000 inhabitants and per year) for regions with lowest OCS use. Red areas indicate highest OCS use, and the green areas indicate lowest OCS use (ranges defined according to the decile distribution)

Although only 4.4% of all patients with OCS prescriptions in 2019 identified in the EMR database corresponded to the patients with severe asthma, the percentage of patients with severe asthma and an OCS prescription during the same year was 27%. The heat map drawn with cold and hot areas regarding the use of OCS in severe asthma is presented in Fig. 2. Regions with the highest OCS use were Asturias, Andalucía (including the autonomous cities Ceuta and Melilla) and Galicia (Fig. 3). Regions with the lowest OCS use were Navarra, Baleares, Madrid and País Vasco (Fig. 4). The estimation of the use of OCS (mg) for 2019, in patients with severe asthma per 100,000 inhabitants for each region was obtained by grouping the basic healthcare areas as presented in the Table 2. By applying the percentage of patients with severe asthma in the general population (0.5%), and the percentage of those who have at least one OCS prescription (27%) to the total OCS use, the mean OCS use per patient in Spain was estimated at 1099.8 mg. Applying the same method, the mean OCS use per patient for the year 2019 was calculated for each region as presented in Table 2.

**Table 2:** Estimation of the OCS use (mg) in patients with severe asthma per 100,000 inhabitants, and estimation of the mean OCS use (mg) per patient with severe asthma and with at least one OCS prescription by Spanish region in 2019

Region	mg/100,000 inhabitants		mg/patient
	Mean	SD	Mean*
Spain	148,479	62,024	1099.85
Andalucía, Ceuta and Melilla	185,682	84,517	1375.42
Aragón	139,255	46,719	1031.52
Asturias	193,406	66,244	1432.64
Cantabria	151,612	54,022	1123.05
Castilla y León	125,784	56,933	931.74
Castilla-La Mancha	138,607	47,553	1026.71
Cataluña	152,360	52,790	1128.59
Comunidad de Madrid	118,311	55,461	876.38
Comunidad Valenciana	141,954	47,347	1051.51
Extremadura	160,772	46,520	1190.90
Galicia	177,671	50,420	1316.08
Islas Baleares	113,766	37,946	842.71
Islas Canarias	145,379	48,979	1076.88
La Rioja	127,735	36,464	946.18
Murcia	151,664	45,337	1123.43
Navarra	105,703	32,111	782.99
País Vasco	120,155	47,445	890.04

*No standard deviation could be calculated because of the methodology used to obtain this figure as explained in methods section: total OCS use during 2019/reference population *0.5% (patients with severe asthma) *27% (patients who had at least one OCS prescription)

## Discussion

This study revealed existence of large geographic differences in OCS use in patients with severe asthma among the different Spanish regions, including the basic healthcare areas of the same region (see Fig. 2). Basic healthcare areas with high OCS use in patients with severe asthma (red areas in the maps) were mainly distributed in Galicia, Asturias, Andalucía, Extremadura, Cataluña, Murcia and Cantabria. By grouping the basic healthcare areas mean, OCS use associated with severe asthma was obtained for each region as shown in Table 2. The analysis further showed that the regions with higher mean OCS use were Asturias, Andalucía (with Ceuta and Melilla), Galicia, Extremadura, Murcia, Cantabria and Cataluña. A plausible explanation for the geographical differences could be the different regional healthcare organization related to asthma management. However, a closer look at the distribution of specialized asthma units across Spanish regions revealed no evident differences [28, 29]. Among regions with the lowest OCS use: Madrid has 1 specialized unit per 500,000 inhabitants, País Vasco 1 per 700,000 and Navarra 1 per 650,000, whereas, Baleares has 1 per 1,100,000 inhabitants. Among regions with the highest OCS use: Asturias has no specialized units, Andalucía has 1 per 1,700,000 inhabitants, and Galicia has 1 per 540,000 inhabitants. Similarly, another recently published study [30] has shown that the prescription of monoclonal antibodies for severe uncontrolled asthma in Spain significantly varies by region, hospital level, and the accreditation level of the asthma units. This study also highlighted the under-prescription of these more effective treatments in Spain. Another possible explanation for geographical differences may be the different number of specialists in pulmonology and allergy in each region. According to the RECALAR study [31], Islas Canarias has only 2.6 specialists per 100,000 inhabitants, Andalucía 2.8 and Cataluña 3.0; whereas, Asturias has 6.0, País Vasco 4.7 and Navarra 4.4. Differences in the level of training of General Practitioners from each region, and in the degree of coordination between primary care and specialized care could also influence the geographical differences found in the present study. Finally, the presence of certain allergens in the air could also have an influence on the higher OCS use in Asturias, Galicia and some areas of the south of Spain. Another study recently published in Spain has shown a heterogeneity between prescribing OCS by primary care physicians, pulmonologists, and allergists [32]. OCS were prescribed mainly in primary care (59%), allergy (13%), and pulmonology (20%). The authors also found that the frequency of prescription of OCS had a direct impact on the main associated adverse effects. Another interesting piece of data from the same study is the analysis of OCS consumption by year in the period from 2015 to 2019. The data showed that between 31.4 and 39.6% of the overall population of patients with asthma had received at least one cycle of OCS depending on the year analyzed [32].

Results presented through the heat maps provide useful information on the specific geographic areas that would especially benefit from OCS-sparing strategies.

According to the data analysed on the *EMR database*, the percentage of patients with severe asthma that had an OCS prescription during 2019 was 27%. This value was similar to the numbers obtained from a previously published study for patients with severe uncontrolled asthma that were treated with OCS for 3 months or longer [25].

The national mean (SD) of OCS use in 2019 per 100,000 inhabitants in Spain was 148,479 (62,024) in mg, amounting to 1099.8 mg per patient with severe asthma and at least one prescription of OCS. This translates to a considerable amount of OCS used, although, it was not possible to estimate if the figure was distributed over shorts courses or prescribed as maintenance treatment. Nevertheless, use of such a high dose of OCS indicates poor asthma control as per a recently published international consensus. According to the consensus, over 75% of experts selected a threshold of 0.5 g or 1 g as the annual cumulative OCS dose indicative of poor asthma control [9], suggesting that higher the cumulative annual OCS dose the greater is the risk of serious adverse effects [10].

Patients with severe asthma selected from the *EMR database* were mostly middle-aged female with a mean BMI over 30 indicating obesity. It was also observed that a significant percentage of patients were active smokers (10%) despite having severe asthma. The median time since diagnosis (close to 7 years) showed that the patients had been living with asthma for a long time. Frequent comorbidities experienced by this group of patients were rhinitis, respiratory infections and chronic sinusitis atopy present in one-third of the patients.

A marked limitation of the study necessitated the use of two different existing databases to build the groups of patients for analysis and therefore, failing to establish comparisons between the groups. This type of analysis in a single and comprehensive database with data of severe asthma patients treated with OCS would have been the best scenario to ensure the highest accurateness. Nevertheless, up to our knowledge, no patient-level database containing this information and with geographical granularity is available in Spain, and therefore this exercise must be just considered as a first piece to build evidence. However, the databases were adequately described to show the advantages and the limitations of their use, and the assumptions and methodology applied were also thoroughly explained in the “Methods” section. Among the advantages of the EMR database are its fairly good representativeness of the overall treated population in Spain and its similar distribution of age and gender compares well to the Spanish general population, but the percentage of Spanish population covered (~ 3.2% of the overall treated population in Spain) could be considered as low. Although the Sell-in database provides information about dispensed prescriptions and does not collect actual intake by the patients, its main advantage is its degree of granularity so that drug use can be analysed at a very detailed degree (basic healthcare areas). Though, the generalizability of the results is limiting, it helps to demonstrate the differences in the OCS use in patients with severe asthma across Spain. One of the major advantages of our study is that it draws attention of the healthcare community towards the high use of systemic corticosteroids in patients with severe asthma in Spain, despite the availability of effective treatments that can help to reduce or even avoid their use.

## Conclusions

There are geographical differences between Spanish regions with respect to the use of OCS in patients with severe asthma. These differences also exist within each region and within each province of a region. At the national level, the use of OCS is higher than the values considered appropriate by the scientific community, and as it is well known OCS use is related to severe adverse events and poor asthma control. It seems necessary to pay special attention to the excessive consumption of OCS in patients with severe asthma and to implement strategies in the Spanish National Health System aimed at reducing OCS consumption in patients with severe asthma.

## Data Availability

The data that support the findings of this study are available from IQVIA, but restrictions apply to the availability of these data, which were used under license for the current study, and so are not publicly available. Data are however available from the authors upon reasonable request and with permission of IQVIA.
